# EIF3C Promotes Lung Cancer Tumorigenesis by Regulating the APP/HSPA1A/LMNB1 Axis

**DOI:** 10.1155/2022/9464094

**Published:** 2022-09-14

**Authors:** Xiaoli Ding, Lanlan Hou, Huijuan Zhang, Zhiping Chen, Zhanyu Liu, Junjie Gong, Zhixian Tang, Rong Hu

**Affiliations:** ^1^Department of Clinical Examination, First Affiliated Hospital of Gannan Medical University, Ganzhou, Jiangxi 341000, China; ^2^Gannan Medical University, Ganzhou, Jiangxi 341000, China; ^3^Thoracic Surgery, First Affiliated Hospital of Gannan Medical University, Ganzhou, Jiangxi 341000, China

## Abstract

**Objective:**

This study was designed to explore the role and mechanism of eukaryotic initiation factor 3C (EIF3C) in the proliferation and apoptosis of lung cancer cells.

**Methods:**

EIF3C expression in clinic lung cancer tissues was detected by immunohistochemistry assay. Cell transfection with lentivirus EIF3C short hairpin RNA (shRNA) was performed with Lipofectamine 2000. Cell proliferation was evaluated by Celigo and MTT assays. Caspase-3/7 activity was assessed using caspase-3/7 assay kit for cell apoptosis detection. The apoptosis rate of lung cancer cells was assessed by flow cytometry. A transplanted tumor nude-mouse model was established to clarify the role of EIF3C in lung cancer. The potential mechanism of EIF3C was explored by mRNA microarray analysis. Among the top 30 up- and downregulated mRNAs selected for RT-qPCR, 5 were chosen for western blot analysis.

**Results:**

EIF3C was abnormally overexpressed in lung cancer cell lines and tissues. Silencing EIF3C suppressed the proliferation and promoted the apoptosis of lung cancer cells. *In vivo* experiments using transplanted tumor nude-mouse model suggested that EIF3C promoted lung cancer tumorigenesis. Further, mRNA microarray analyses identified 189 upregulated and 83 downregulated differentially expressed mRNA between the KD and negative control groups. After validation by RT-qPCR and western blot, three downstream genes (APP, HSPA1A, and LMNB1) were confirmed.

**Conclusion:**

EIF3C overexpression may facilitate the proliferation and hamper the apoptosis of lung cancer cells by regulating the APP/HSPA1A/LMNB1 axis.

## 1. Introduction

In the past decades, lung cancer has become the most common malignancy and the major cause of deaths related to cancer worldwide [1]. The widely applied therapy managements for lung cancer include surgical resection, chemoradiotherapy, target treatment, and immunotherapy. However, the overall survival time of patients with lung cancer remains poor at <20% 5-year overall survival rate [2]. Thus, exploring the detailed molecular mechanisms underlying the pathological progression of lung cancer and identifying novel biomarkers is necessary for the early diagnosis, prevention, and treatment of this disease.

Eukaryotic translation initiation factor (EIF) is critical in eukaryotic translation initiation. At present, 12 kinds of EIF have been found [3]. Among which, EIF3C is considered to be a house-keeping gene. Many researchers believe that the assembly of the EIF3 complex is necessary for the cytoplasm [4, 5]. EIF3C is abnormally expressed and exerts an important regulatory role in various human cancer physiological processes, such as cell proliferation and tumorigenesis [6]. For instance, EIF3C can enhance hepatocellular carcinoma cell proliferation in vitro and tumor growth in vivo [7]. EIF3C knockdown in HCT116 cells can significantly hamper cell proliferation and cause G2/M phase arrest [8], and its knockdown in pancreatic cancer cells could suppress cell proliferation and accelerate apoptosis [9]. However, the role and specific regulatory mechanism of EIF3C in lung cancer tumorigenesis have not been fully studied.

This study was designed to explore the role and mechanism of EIF3C in lung cancer proliferation and apoptosis to provide new therapeutic biomarkers for predicting the progression of this disease.

## 2. Methods

### 2.1. Clinical Sample Collection

Tumors and adjacent carcinoma tissues were obtained under surgery from 117 patients with lung cancer and rapidly frozen in liquid nitrogen until use. All patients provided informed consent, and this work was approved by the Ethics Committee of the First Affiliated Hospital of Gannan Medical University.

### 2.2. Cell Culture

Lung cancer cell lines (H1299, A549, H1975, and H1688) were purchased from the Cell Bank of Chinese Academy of Sciences. After being resuscitated, the cells were maintained in RPMI-1640 medium containing 10% FBS in a routine condition. Cells at 70%–80% confluence were used for transfection experiments.

### 2.3. Plasmid and Cell Transfection

Lentivirus EIF3C short hairpin RNA (shRNA, 5′-GACCATCCGTAATGCCATGAA-3′) and its negative control (NC, 5′-TTCTCCGAACGTGTCACG-3′) both containing GFP were purchased from Shanghai GeneChem Co., Ltd. The cells were maintained overnight to 80% confluence, and cell transfection was performed with Lipofectamine 2000 (Invitrogen) following the manufacturer's recommended protocols.

### 2.4. Cell Proliferation

The cells were digested by trypsin, suspended in a complete medium, seeded at 5 × 10^4^ cells/well (three wells per group), and incubated at 37°C with 5% CO_2_ for 24 h. After transfection, the cell number was counted using Celigo-based method once a day for 5 consecutive days [10]. The GFP-positive cells were identified and scanned by Celigo image cytometry. The image captured by the image cytometer was equivalent to 100× of the microscope, and the cell growth curve was plotted.

### 2.5. Quantitative Real-Time PCR (qRT-PCR) Assay

Total RNA of different cells was extracted by the Trizol method (Invitrogen, CA, USA). cDNA was reversely transcribed from RNA (1 *μ*g) using Prime Script RT Master Mix kit. PCR was performed with StepOnePlus™ Real-Time PCR System. The primer sequences are shown as follows: SPP1, forward: five groups were expressed as an average captured by image cytometer Co., Ltd. Co., Ltd. ting the progression insulation (15). reverse: 5′-CGTTGGCGGAGTGGATGT-3′; amyloid precursor protein (APP), forward: 5′-CTGATGCGGAGGAGGATGAC-3′; reverse: 5′-TCTCTGTGGCTTCTTCGTAGG-3′; RAP1A, forward: 5′-CGTGAGTACAAGCTAGTGGTCC-3′; reverse: 5′-CCAGGATTTCGAGCATACACTG-3′; lamin B1 (LMNB1), forward: 5′-AATGGGAGGCTGGGAGATGAT-3′; reverse: 5′-TGGTTCTTCCAGATGAGGTCAGTT-3′; ERO1B, forward: 5′-TTCTGGATGATTGCTTGTGTGAT-3′; reverse: 5′-GGTCGCTTCAGATTAACCTTGT-3′; PRKAA2, forward: 5′-TGATGATGAGCATGTACCTAC-3′; reverse: 5′-CGACAGAACGATTGAGATATTC-3′; MAPK9, forward: 5′-AGCCAACTGTGAGGAATTATGT-3′; reverse: 5′-GCTTGTCAGGATCAATCACTAAC-3′; DDIT3, forward: 5′-GAACCAGGAAACGGAAACAG-3′; reverse: 5′-ATTCACCATTCGGTCAATCA-3′; heat shock protein family A member 1A (HSPA1A), forward: 5′-CAAGACTTTGCATTTCCTAG-3′; reverse: 5′-GGCCTGAGTTAAGTGTATT-3′; PPP2R5A, forward: 5heAAGAACACTGGAATCCGACCA-3′; reverse: 5′-TTCGGCACTTGTATTGCTGAG-3′; SEPT2, forward: 5′-ACCTCCCCAATCAAGTTCACC-3′; reverse: 5′-TAGCTTGACCCCTCGCTCTTC-3′; and GAPDH, forward: 5′-TGACTTCAACAGCGACACCCA-3′; reverse: 5′-CACCCTGTTGCTGTAGCCAAA-3′. GAPDH was used as an internal control.

### 2.6. Western Blot

Total proteins (30 *μ*g) were extracted from the cultured cells. Each protein sample was isolated with 10% SDS-PAGE and transferred to a nitrocellulose membrane. The membranes and primary antibodies were then incubated overnight at a dilution of 1 : 1,000 under 4°C. Antibodies against DDIT3 (Abcam, AB11419), APP (CST, #2452), HSPA1A (CST, #4872), LMNB1 (CST, #12586), RAP1A (Abcam, AB96223), and GAPDH (SANTA CRUZ, sc-32233) were used as the primary antibodies. HRP-conjugated antimouse immunoglobulin G (cst, #7076; 1 : 2, 000) and antirabbit immunoglobulin G (cst, #7074; 1 : 2, 000) were used as the secondary antibodies. Protein bands were visualized using Chemiluminescence ECL Kit (Millipore).

### 2.7. Caspase3/7 Activity Assay

Cell apoptosis as reflected by caspase3/7 activity was detected by a caspase 3/7 assay kit (Sangon, Biotech) according to the manufacturer's protocol. The lung cancer cells were seeded in 96-well plates, incubated with a completed culture medium for a certain period, collected by centrifugation, and resuspended in the RIPA buffer. The lysate was mixed with the reagent with a ratio of 1 : 1. Caspase3/7 activity was evaluated by measuring luminescence with a luminometer.

### 2.8. Flow Cytometry Analysis

The apoptosis rate of cells post 48-h transfection was detected by flow cytometry. For cell apoptosis, the cells were seeded in 96-well plates at 2 × 10^5^/well and incubated with 5 *μ*L of Annexin V-FITC and 10 *μ*L of PI for 10 min. The percentage of apoptotic cells was detected by flow cytometry.

### 2.9. In Vivo Imaging

Biological distribution was monitored using near-infrared dye Alexa fluor 633 coupled to bovine serum albumin carrier. An intravenous injection of 200 *μ*L of sh-EIF3C was performed on mice for 24 h. After the mice were anesthetized, the small animal VIS imaging spectroscopy system was equipped with fluorescent filters (excitation emission, 633/647 nm) and was used to capture fluorescent images, which were normalized and reported as counts per second (counts/second).

### 2.10. Immunohistochemistry Assay

Tissue sections (5 *μ*m) were deparaffinized, antigen repaired, and sealed with1.5% goat serum for 30 min at 37°C. The slides were treated with primary antibody against EIF3C (1 : 1000 dilution) overnight and biotin-conjugated goat antirabbit immunoglobulin G secondary antibody with 1 : 1, 000 dilution. The target protein was stained with DAB solution and observed under a microscope.

### 2.11. Xenograft Model

Twenty Balb/c nude mice were randomly assigned to KD and NC groups (*n* = 10 per group), which were subcutaneously injected with 100 *μ*L of H1299 cells (2 × 10^6^) transfected with sh-EIF3C and sh-NC, respectively. The mice were maintained in a routine condition for 5 consecutive weeks. Tumor volume (length × width) was monitored once a week. At the end of 5 weeks, the mice were sacrificed, and the tumor weight was recorded. The animal experiments were conducted in compliance with animal care guidelines and had received the Ethics committee approval.

### 2.12. mRNA Microarray and Data Analyses

H1299 cells stably expressing sh-EIF3C and sh-NC were subjected to mRNA microarray analyses (*n* = 3 per group). The efficiency of sh-EIF3C transfection is shown in Figure S1. After the raw microarray data were preprocessed, differentially expressed genes (DEGs) were identified with *p* < 0.05 and |FC| > 1.5 and subjected to hierarchical clustering analysis. The expression of the top 30 DEGs was detected by RT-qPCR analysis. Among these DEGs, 5 genes of interest were further detected by western blot analysis. Ingenuity pathway analysis (IPA, http://www.ingenuity.com) and disease and function annotation with the R package were executed to understand the function of these DEGs.

### 2.13. Statistical Analysis

Independent experiments were performed in three replications, and the data were represented as mean ± SD. Statistical analysis of all data was conducted with SPSS 21.0. Student's *t*-test was used for comparison between two groups, and one-way ANOVA and Tukey's posttests were applied for multiple groups. The *p* < 0.05 was set as the significance level.

## 3. Results

### 3.1. EIF3C Is Overexpressed in Lung Cancer Tissues and Cell Lines

**Figure 1:**
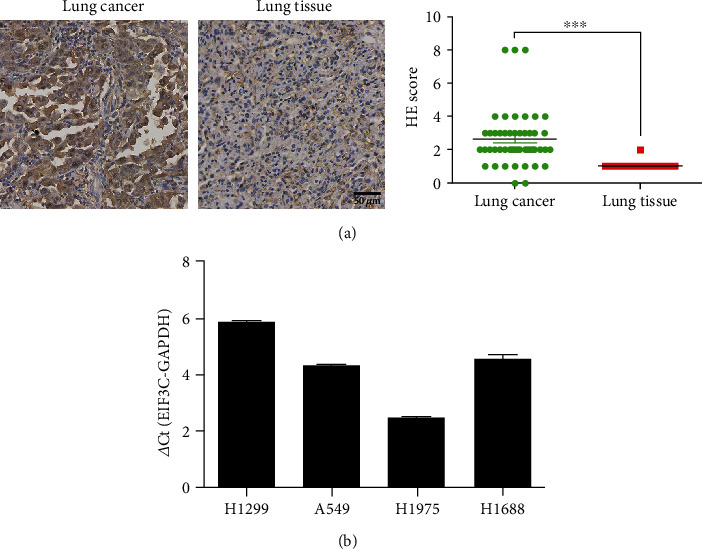
EIF3C is highly expressed in lung tissues and cell lines. (a) The expression of EIF3C in lung cancer detected by immunohistochemistry. Significant elevation was observed in EIF3C expression in tumor tissues compared with adjacent normal tissues collected from 117 lung cancer patients. (b) The expression of EIF3C in lung cancer cells investigated by qRT-PCR assay. ^∗∗∗^*p* < 0.001 compared with control. The experiments were repeated three times (*n* = 3).

EIF3C expression in clinic lung cancer tissues was detected using immunohistochemistry.  Figure 1(a) shows that EIF3C was significantly upregulated in lung tumor tissues compared with that in normal lung tissues (*p* < 0.001). EIF3C expression in lung cancer cells was further evaluated by qRT-PCR assay.  Figure 1(b) indicates that EIF3C expression was the highest in H1299 cells, followed by that in H1688, A549, and H1975 cells. Therefore, H1299 cells were chosen for further study. All of these findings suggested that EIF3C is abnormally expressed in lung cancer tissues and might play a significant function in lung tumorigenesis.

### 3.2. EIF3C Promotes the Proliferation and Inhibits the Apoptosis of Lung Cancer Cells

**Figure 2:**
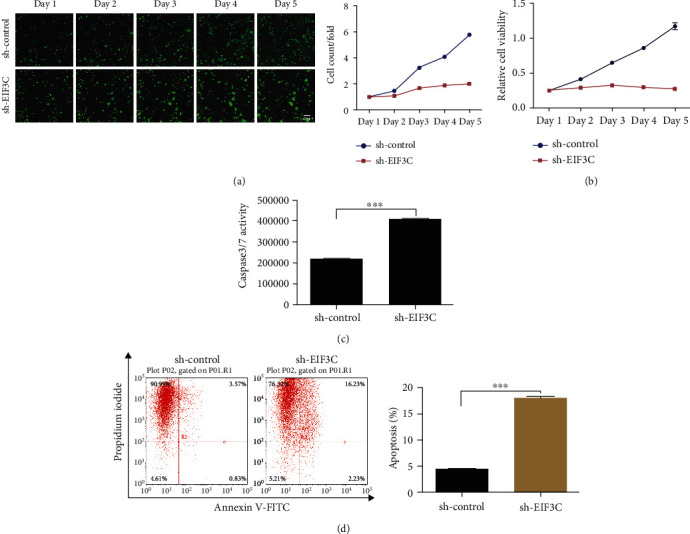
EIF3C promotes the proliferation and inhibits apoptosis of lung cancer cells. (a) The number of cells at 5 days after cell transfection detected by the Celigo method. sh-EIF3C dramatically suppressed the proliferation of lung cancer cells. (b) Cell proliferation after EIF3C silencing measured by MTT. The inhibitory effect of sh-EIF3C was also observed in the MTT. (c) The activity level of caspase-3/caspase-7 after cell transfection. sh-EIF3C significantly promoted the activity of caspase-3/caspace-7. (d) The apoptosis of lung cancer cells assessed by flow cytometry assay. sh-EIF3C significantly accelerated the apoptosis of lung cancer cells. ^∗∗∗^*p* < 0.001 compared with control. The experiments were repeated three times (*n* = 3).

The effect of EIF3C on lung cancer progression was measured by a loss-function experiment. H1299 cells were transfected with sh-EIF3C or NC and subjected to Celigo and MTT assays. The results showed that low EIF3C expression resulted in decreased cell count and viability (Figures 2(a) and 2(b)). Caspase-3/7 activity was assessed to evaluate cell apoptosis, and our data exhibited that caspase-3/7 activity was significantly elevated in the cells treated with sh-EIF3C (*p* < 0.001, Figure 2(c)). Flow cytometry assay indicated that low EIF3C expression enhanced the apoptosis of lung cancer cells (*p* < 0.001, Figure 2(d)).

### 3.3. EIF3C Promotes Lung Cancer Tumorigenesis In Vivo

**Figure 3:**
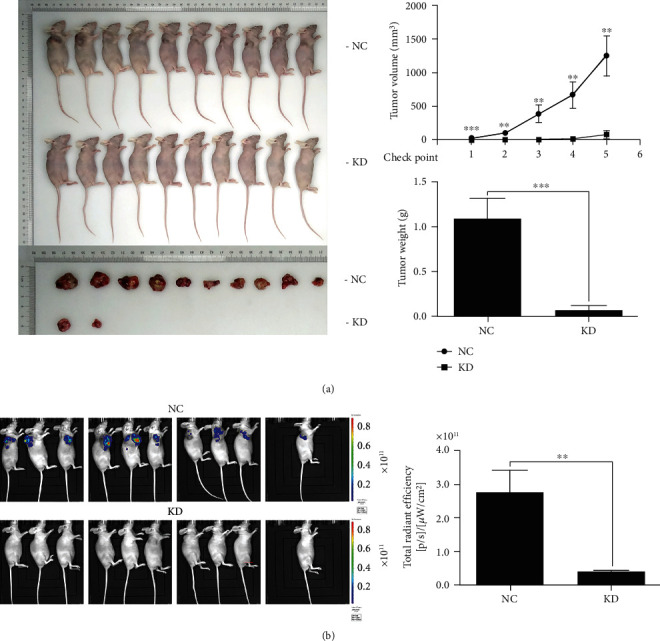
EIF3C promotes tumorigenesis of lung cancer in vivo. (a) Tumor volume and tumor weight in nude mice transplanted with tumor. sh-EIF3C suppressed the growth of tumors with time in mice. (b) Near-infrared fluorescence imaging of intact mice at 24 h after intravenous injection of sh-EIF3C. sh-EIF3C dramatically suppressed the radiant efficiency of tumor, and no fluorescence was observed in after the injection of sh-EIFC3. ^∗∗^*p* < 0.01, ^∗∗∗^*p* < 0.001 compared with control. The experiments were repeated three times (*n* = 3).

A transplanted tumor nude-mouse model was established to clarify the role of EIF3C in lung cancer in vivo. The results indicated that EIF3C knockdown reduced tumor growth as reflected by a decline in tumor volume and weight (all *p* < 0.01, Figure 3(a)). Infrared dye Alexa fluor 633-linked sh-EIF3C was injected intravenously into normal mice to determine the presence of the plasmid carrying sh-EIF3C in the mice in vivo. The biological distribution of sh-EIF3C in vivo was measured by fluorescence imaging. Near-infrared fluorescence images of intact mice were obtained 24 h after sh-EIF3C intravenous injection. The results showed that EIF3C knockdown dramatically suppressed the radiant efficiency, but no fluorescence was observed in the KD mice (Figure 3(b)).

### 3.4. mRNA Profile among the Groups

**Figure 4:**
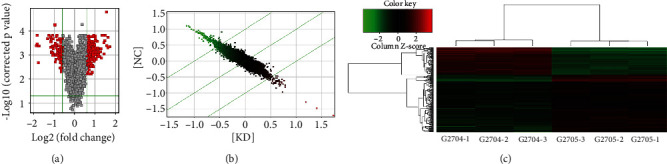
mRNAs profile among the groups. The differential expressed miRNAs between the two groups of samples were identified and visualized by volcano plots (a), scatter plots (b), and hierarchical clustering heat maps (c). The experiments were repeated three times (*n* = 3).

**Figure 5:**
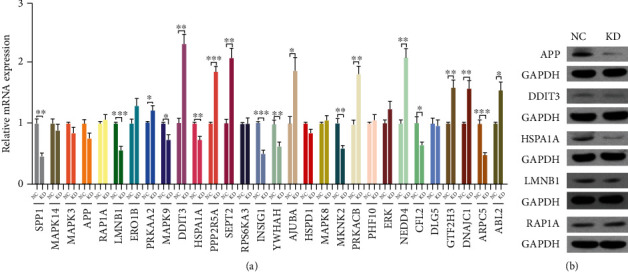
mRNA and protein expression levels of key genes. (a) The expression of top 30 up- and downregulated mRNAs detected by RT-qPCR. (b) The relative protein expressions of DDIT3, APP, HSPA1A, LMNB1, and RAP1A analyzed by western blot assay. ^∗^*p* < 0.05, ^∗∗^*p* < 0.01, ^∗∗∗^*p* < 0.001 compared with control.

A total of 272 mRNAs with differential expression were detected between the KD and NC groups; among which, 189 were upregulated and 83 were downregulated. The differential expression of genes between the two groups was visualized by volcano plots (Figure 4(a)), scatter plots (Figure 4(b)), and heat map (Figure 4(c)). The top 30 mRNAs with differential expression were selected for subsequent analyses to validate the microarray results. Gene expression detected by RT-qPCR is illustrated in Figure 5(a). Moreover, the relative protein expression of DDIT3, APP, HSPA1A, LMNB1, and RAP1A was measured by western blot assay. The results showed the differential expression of APP, HSPA1A, and LMNB1 at the protein level (Figure 5(b)), which was consistent with the microarray findings.

**Figure 6:**
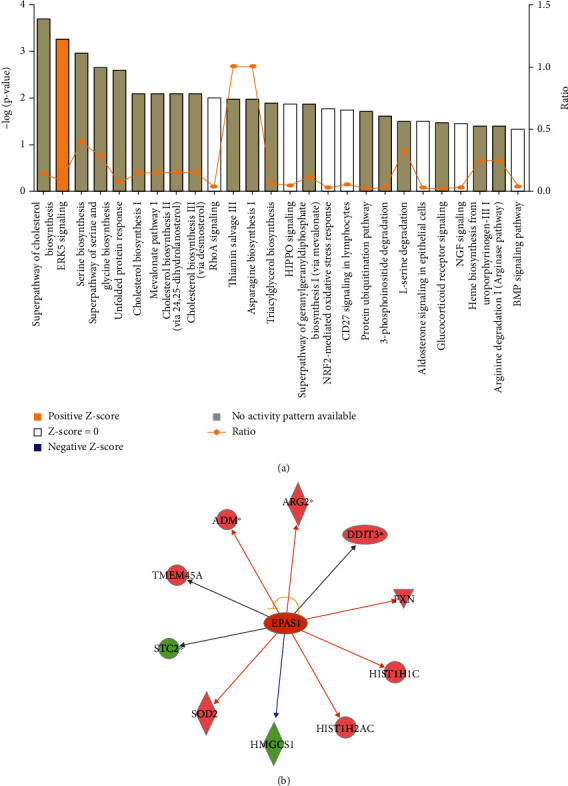
Canonical pathways enriched by DEGs based on IPA. (a) Significant pathways closely related with DEGs. (b) Regulatory network of upstream EPAS1 with DEGs. The orange line represents activated status, blue line represents inhibitive status, yellow line means different expression status, and gray line means no regulatory interaction.

**Figure 7:**
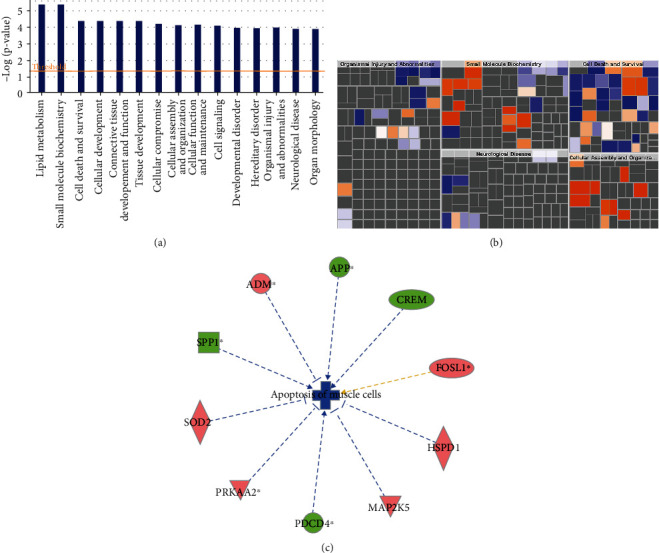
Results of disease and function annotation analysis. (a) The top 15 function categories significantly enriched by DEGs. (b) The heat map of disease and function affected by DEGs. Orange, *z* score>0, function is activated; blue, *z* score< 0, function is inhibited; gray, no *z* score value. (c) Regulatory network of function with DEGs. Blue line means inhibition; orange line means activation.

IPA and function enrichment analysis were performed to explore the biological function of DEGs. Twenty-six canonical pathways closely associated with DEGs are depicted in Figure 6(a), among which, ERK5 signaling (*z* score = 0.447) was the most significant activated pathway. The interactions between molecules in ERK5 signaling are illustrated in Figures S2 and S3. On the basis of the activation *z* score algorithm of IPA, EPAS1 was predicted to be the upstream regulator that is strongly activated by DEGs. The upstream regulatory network showed that EPAS1 had direct interaction with 10 DEGs. The detailed regulatory interactions are depicted in Figure 6(b). According to the disease and function annotation analysis, the DEGs were significantly enriched in lipid metabolism, small molecule biochemistry, cell death, and survival related function categories (Figure 7(a)). The DEGs activated glucose tolerance (*z* score = 2.209) and the movement of organelles (*z* score = 2.193) but suppressed the apoptosis of muscle cells (*z* *s*core = −2.505) and heart (*z* score = −2.303) (Figure 7(b)). The apoptosis of muscle cells was the most significantly inhibited, and the regulatory interactions of DEGs are listed in Figure 7(c).

## 4. Discussion

The occurrence and development of human cancers involve a long-term complex process mainly due to a series of abnormalities occurring at the molecular level, such as the mutations of genes, the abnormal inactivation or activation of some signaling pathways, and the imbalance of homeostasis in the cell environment [11]. In recent years, an increasing number of studies have focused on investigating the genes associated with the proliferation and apoptosis of lung cancer cells to promote the understanding of the molecular mechanisms underlying the development of this malignancy [12–14].

The human EIF3 complex is composed of 13 subunits, and its functional core consists of six subunits; EIF3a, EIF3b, and EIF3c are conserved in all eukaryotes [15]. EIF3 can bind to the mRNAs associated with cell proliferation to control their translation [16]. Thus, studies about EIF3 are mainly concentrated on cancers [7, 17]. EIF3C was found to be upregulated in pancreatic cancer, and its knockdown significantly suppressed cell proliferation and enhanced cell apoptosis [18]. In the present study, EIF3C was found to be abnormally overexpressed in lung cancer tissues. Silencing EIF3C hampered the proliferation and promoted the apoptosis of lung cancer cells. *In vivo* experiments using a transplanted tumor nude-mouse model suggested that EIF3C promoted lung cancer tumorigenesis. Further mRNA microarray analyses identified 189 upregulated and 83 downregulated differentially expressed mRNAs between the KD and NC groups. After validation by RT-qPCR and western blot, three downstream genes (APP, HSPA1A, and LMNB1) were confirmed.

Some EIF3 subtypes have been suggested to influence cancer prognosis by affecting the proliferation and apoptosis of tumor cells [19]. ZNF280A may promote the development of lung adenocarcinoma by interacting with EIF3C, thus implying that EIF3C may have a potential role in lung cancer [12]. EIF3C is upregulated in renal cell carcinoma [20] and intrahepatic cholangiocarcinoma [21]. In our study, we first demonstrated that EIF3C was overexpressed in lung cancer. In addition, EIF3C could facilitate the proliferation and inhibit the apoptosis of lung cancer cells and promote the tumorigenesis of lung cancer.

We then performed mRNA microarray analyses to further investigate the possible mechanisms of the tumorigenic effect of EIF3C. Microarray data can provide a functional molecular understanding of lung cancer, insights into its pathophysiology, and relevant information for therapeutic decisions [22]. We identified 189 upregulated and 83 downregulated differentially expressed mRNAs between the KD and NC groups. We selected the top 30 up- and downregulated mRNAs for RT-qPCR. Among these mRNAs, we further selected five ones for western blot analysis. The expression patterns of APP, HSPA1A, and LMNB1 were verified to be consistent with the microarray data.

APP is a single-channel transmembrane involved in a variety of cellular functions [23]. Together with its two homologs (APLP-1 and APLP-2), this protein is associated with various cell functions, such as iron transport and homeostasis [24]. Some studies have suggested that APP is implicated in tumorigenesis by activating signal-regulated protein kinase in cells [25]. Strilic et al. [26] reported that tumor cells can promote hematogenous metastasis through APP upregulation. Furthermore, the APP intracellular domain can interact with adaptor proteins to activate downstream signaling molecules, thereby affecting the metastasis of tumor cells [27]. A recent study revealed that APP can promote the aggression of breast cancer cells by activating the MAPK signaling [28] and is upregulated in lung cancer [29]. In the present work, APP was significantly downregulated in the EIF3C knockdown group. Thus, we speculated that EIF3C may promote lung cancer tumorigenesis by regulating APP.

HSPA1A belongs to the heat shock protein 70 family and mediates the folding of the new translation protein [30]. This protein is upregulated in many types of cancers and contributes to tumor cell proliferation and decreased cancer cell apoptosis [31, 32]. High HSP70 expression is associated with poor lung cancer prognosis [33]. HSPA1A also plays a role in the PI3K/AKT signaling pathway [34]. Cheng et al. [34] reported that MnSO_4_ exposure could activate the PI3K/AKT pathway in the hippocampus of rats and subsequently increase HSPA1A transcription and translation [34]. Nevertheless, whether EIF3C promotes lung cancer tumorigenesis by regulating HSPA1A via the PI3K/AKT pathway warrants further investigation.

LMNB1 is a member of a lamin protein family and an important component protein of the nuclear skeleton [35]. It regulates cell proliferation, DNA replication, and DNA damage repair and maintains nuclear integrity [36]. Therefore, the aberrant expression of this gene plays a critical role in carcinogenesis. LMNB1 is aberrantly highly expressed in lung adenocarcinoma tissues compared with tumor-adjacent tissues, thus regulating the proliferation ability of the lung adenocarcinoma cells via the AKT pathway [37]. Tang et al. [38] suggested that silencing LMNB1 could suppress the development of lung adenocarcinoma. In the present study, LMNB1 was suppressed after EIF3C silencing. Thus, we speculated that EIF3C may play a carcinogenic role in lung cancer by regulating LMNB1.

Studies that further validated the interaction between EIF3C and the predicted genes, especially their regulatory effect on lung cancer progression, are lacking. Several clinical studies reported the significance of EIF3C in predicting patients' prognosis in various human cancers, such as intrahepatic cholangiocarcinoma, prostate cancer, and endometrial cancer [21, 39, 40]. However, the significance of EIF3C and its potential correlated genes in the prognosis and disease development of lung cancer has not been revealed in the present study and would be the future direction of our investigations.

In summary, we suggested for the first time that EIF3C is upregulated in lung cancer tissues and may enhance the proliferation and suppress the apoptosis of lung cancer cells by regulating the APP/HSPA1A/LMNB1 axis. EIF3C may serve as a novel therapeutic biomarker in lung cancer.

## Data Availability

The data used to support the findings of this study are included within the article.

## Supplementary Materials

Supplementary Materials Figure S1 The expression of EIF3C as comparison to transfected with NC detected by RT-qPCR. ^∗∗∗^*p* < 0.001 compared with control. Figure S2 Signal transmission of molecules in ERK5 signaling affected by DEGs red, upregulated; green, downregulated. Figure S3 Signal transmission of molecules in ERK5 Signaling supported by published literatures red, upregulated; green, downregulated.
