# Supplementary material for: EIF3C Promotes Lung Cancer Tumorigenesis by Regulating the APP/HSPA1A/LMNB1 Axis
Source: Dis Markers. 2022 Sep 14;2022:9464094. doi: 10.1155/2022/9464094 (PMC9492341; doi:10.1155/2022/9464094)

Figure S1 The expression of EIF3C as comparison to transfected with NC detected by RT-qPCR. ***p < 0.001 compared with control.

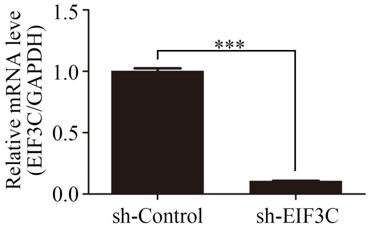

Figure S2 Signal transmission **of molecules in ERK5 Signaling affected by DEGs**

Red, up-regulated; green, down-regulated.

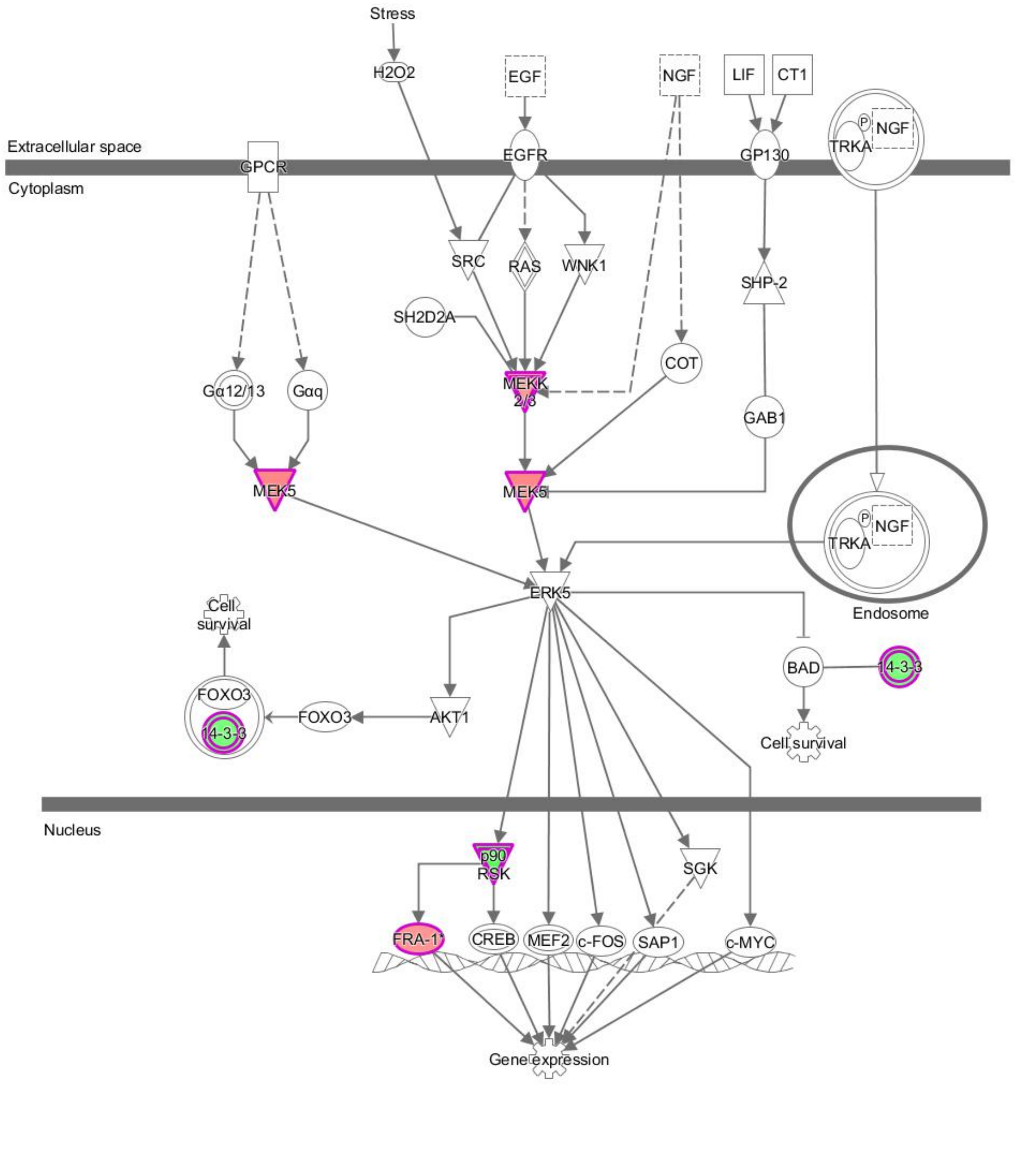

Figure S3 Signal transmission **of molecules in ERK5 Signaling supported by published literatures**

Red, up-regulated; green, down-regulated.

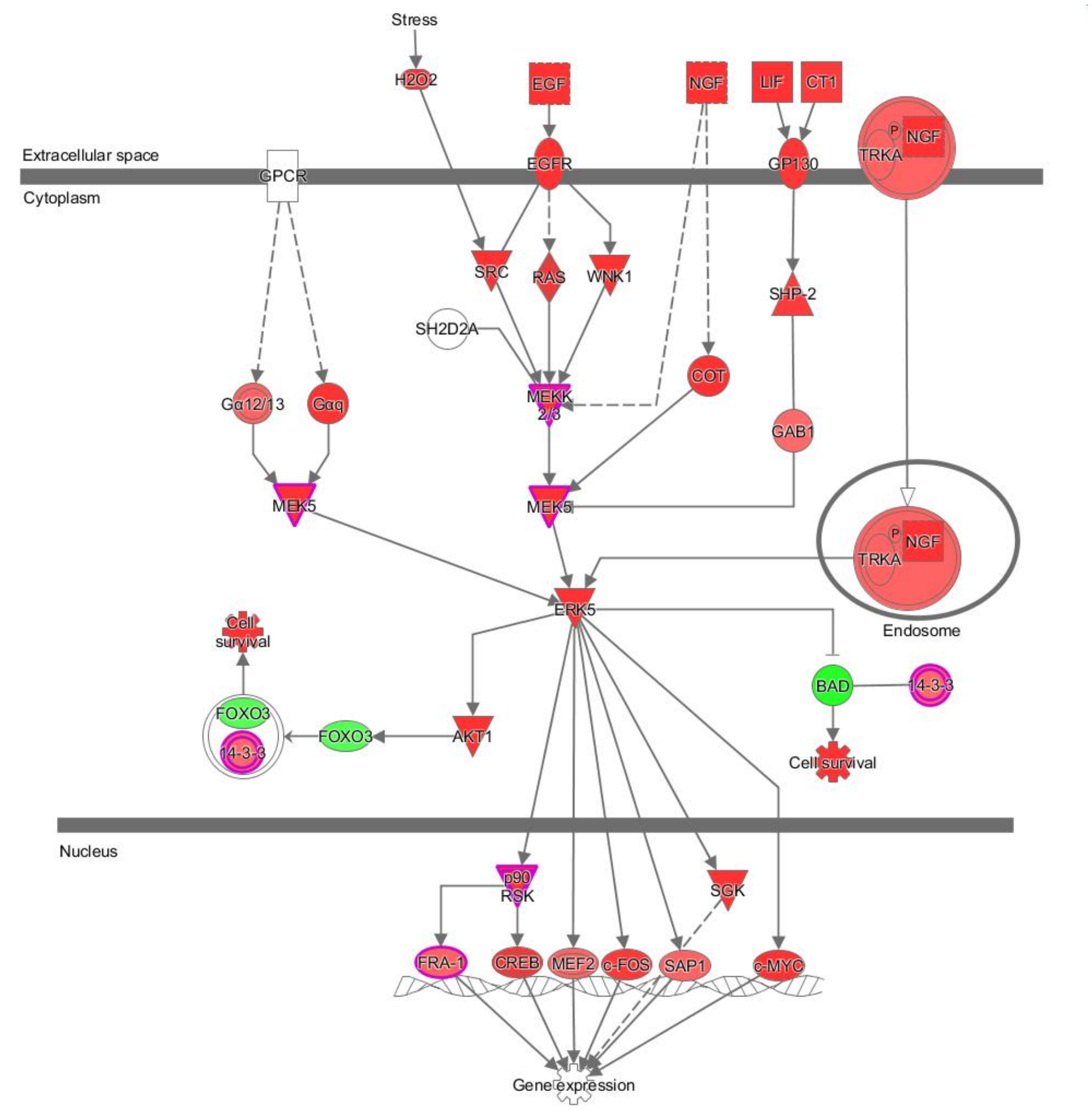

Supplement: Supplementary Materials — Figure S1 The expression of EIF3C as comparison to transfected with NC detected by RT-qPCR. ∗∗∗p < 0.001 compared with control. Figure S2 Signal transmission of molecules in ERK5 signaling affected by DEGs red, upregulated; green, downregulated. Figure S3 Signal transmission of molecules in ERK5 Signaling supported by published literatures red, upregulated; green, downregulated. [file 9464094.f1.pdf]
